# Identification of Potentially Therapeutic Immunogenic Peptides From *Paracoccidioides lutzii* Species

**DOI:** 10.3389/fimmu.2021.670992

**Published:** 2021-05-11

**Authors:** Leandro B. R. Silva, Cleison L. Taira, Levi G. Cleare, Michele Martins, Magno Junqueira, Joshua D. Nosanchuk, Carlos P. Taborda

**Affiliations:** ^1^ Departamento de Microbiologia, Instituto de Ciencias Biomedicas, Universidade de Sao Paulo, Sao Paulo, Brazil; ^2^ Departments of Medicine (Division of Infectious Diseases) and Microbiology and Immunology, Albert Einstein College of Medicine, New York, NY, United States; ^3^ Proteomics Unit, Department of Biochemistry, Chemistry Institute, Federal University of Rio de Janeiro, Rio de Janeiro, Brazil; ^4^ Laboratorio de Micologia Medica (LIM53), Departamento de Dermatologia, Faculdade de Medicina, Instituto de Medicina Tropical de Sao Paulo, Universidade de Sao Paulo, Sao Paulo, Brazil

**Keywords:** paracoccidioidomycosis, vaccine, peptide, *Paracoccidioides lutzii*, PCM

## Abstract

Paracoccidioidomycosis (PCM) is an endemic mycosis in Latin America caused by the thermodimorphic fungi of the genus *Paracoccidioides* spp. *Paracoccidioides lutzii* (PL) is one of the 5 species that constitute the *Paracoccidioides* genus. PL expresses low amounts of glycoprotein (Gp) 43 (PLGp43) and PLGp43 displays few epitopes in common with the *P. brasiliensis* (PB) immunodominant antigen PBGp43, which is commonly used for serological diagnosis of PCM. This difference in structure between the glycoproteins markedly reduces the efficiency of serological diagnosis in patients infected with PL. We previously demonstrated that peptide 10 (P10) from the PBGp43 induces protective immune responses in *in vitro* and *in vivo* models of PB PCM. Since, P10 has proven to be a promising therapeutic to combat PB, we sought to identify peptides in PL that could similarly be applied for the treatment of PCM. PL yeast cell proteins were isolated from PL: dendritic cell co-cultures and subjected to immunoproteomics. This approach identified 18 PL peptides that demonstrated *in silico* predictions for immunogenicity. Eight of the most promising peptides were synthesized and applied to lymphocytes obtained from peptide-immunized or PL-infected mice as well as to *in vitro* cultures with peptides or dendritic cells pulsed the peptides. The peptides LBR5, LBR6 and LBR8 efficiently promoted CD4^+^ and CD8^+^ T cell proliferation and dendritic cells pulsed with LBR1, LBR3, LBR7 or LBR8 stimulated CD4^+^ T cell proliferation. We observed increases of IFN-γ in the supernatants from primed T cells for the conditions with peptides without or with dendritic cells, although IL-2 levels only increased in response to LBR8. These novel immunogenic peptides derived from PL will be employed to develop new peptide vaccine approaches and the proteins from which they are derived can be used to develop new diagnostic assays for PL and possibly other *Paracoccidioides* spp. These findings identify and characterize new peptides with a promising therapeutic profile for future against this important neglected systemic mycosis.

## Introduction

Paracoccidioidomycosis (PCM) is one of the major systemic fungal diseases in Latin America, with the highest incidence occurring in Brazil, Colombia and Venezuela (1–3). PCM is caused by fungi of the genus *Paracoccidioides*, which is constituted by the phylogenetic complex *Paracoccidioides brasiliensis* and the species *P. lutzii* (PL) (4). *The P. brasiliensis* complex was recently molecularly separated as *P. brasiliensis* (PB) and three new species: *P. americana*, *P. restrepiensis* and *P. venezuelensis*  (5, 6). Remarkably, PCM due to each of these 5 *Paracoccidioides* species causes disease manifestations that are indistinguishable from one another.

In Brazil, PCM is not a mandatory reportable disease. Hence, the actual incidence of PCM is unknown, but it is estimated that the annual incidence is increasing from 0.71 to 3.7 cases per 100,000 inhabitants between 1980 and 1999 (7). There are regions with higher rates of PCM, such as in Rondônia were there are 9.4 cases per 100,000 inhabitants, with two cities reporting incidences of ~40 cases per 100,000 inhabitants (8). Although the specific distribution of *Paracoccidioides* species has not been entirely clarified (9–11), Rondônia, Mato Grosso, Pará are states in the north and central-west regions of Brazil where PL has been well documented (4, 12). The true incidence of each *Paracoccidioides* species is difficult to establish in part due to the limited ability to diagnose the infection. The standard diagnostic of PCM requires a direct identification of the fungus in fresh biological material (sputum, lesion scraping or lymph node aspirate examination) followed by isolation of the fungus which is hard and can take more than 15 days for the fungus to grow (13). The glycoprotein Gp43 produced by PB (PBGp43) is one of the main serological markers used in the diagnosis of PCM (14–17). However, PL expresses low amounts of Gp43 and PLGp43 displays few epitopes in common with the immunodominant PBGp43, which markedly reduces the efficiency of serological diagnosis in patients infected with PL (18, 19).

In addition to being applied for serological diagnosis, PBGp43 has been explored as a therapeutic (20). In particular, the peptide 10 (P10) from the PBGp43 (20) induces protective immune responses in *in vitro* and *in vivo* models. P10 induces proliferation of cells T CD4^+^ Th_1_ and the expression of high levels of INF-*γ* and IL-2 (20). Administration of P10 either before or after the establishment of PB PCM produces a therapeutically protective effect in both immunosuppressed and immunocompetent mice (21–24). P10 requires concomitant administration of adjuvant for efficacy although diverse adjuvants produce a therapeutic effect with the peptide (25), including delivery as a P10-nanoparticle (26). However, these protection experiments have been carried out using the PB (Pb18) isolate. PLGp43 is an active glucanase with partial antigenic identify with PBp43 (18), but the specific sequence of P10 has important amino acids changes in the equivalent region in PLGp43. Hence, P10 is unlikely to lead to protection against PL.

Since, P10 has proven to be a promising therapeutic to combat PB PCM, we sought to identify peptides in PL that could similarly be applied for treatment (27, 28). To accomplish this, we used an immunoproteomic approach, which is a powerful tool for identification and characterization of molecules with potential to serve as biomarkers for diagnoses (29–33). We validated select identified peptides for their ability to activate macrophages and dendritic cells as well as their ability to stimulate the proliferation of lymphocytes from mice immunized with the peptides. Of the 8 peptides we identified and synthesized, 4 promoted the proliferation of CD4^+^ T cells in co-culture with dendritic cells. Additionally, 2 and 3 peptides were able to promote the proliferation of CD4^+^ and T CD8^+^ T cells, respectively, when the peptides were added to culture medium. These findings support the continued pursuit of immunogenic peptides as therapeutics to combat PL PCM.

## Material and Methods

### Fungal Strains

PL (Pb8334) was grown in Fava-Netto solid medium at 37°C for 7 days. Thereafter, cells were isolated and washed three times in PBS, and large and agglomerated yeast cells were discarded resulting in the collection of uniform small yeast cells. Yeast cells were then heat inactivated at 60°C for 2 hours.

### Mice

All procedures were performed according to the guidelines of National Council of Ethics with Animals (CONCEA) and the protocols were approved by the Ethical Committee for Animal Use from Institute of Biomedical Sciences at University of Sao Paulo (CEUA N° 2185220219, approved in 04/28/2019) and at Albert Einstein College of Medicine (Einstein) under the animal use protocol n° 00001281.

### Culture Cells

Bone Marrow-derived Dendritic Cells (BMDC) were generated using 6-8-week-old male BALB/c mice according to an established protocol (22, 34). Briefly, mice were euthanized, and their femurs and tibias removed and flushed with RPMI (Vitrocell, Campinas, Brazil) medium to release bone marrow cells. The cells were counted in a hemocytometer and were plated at 10^7^ cells/plate/10ml in 90mm × 15mm Petri with RPMI supplemented with IL-4 (15ng/ml) (Invitrogen), rGM-CSF (30ng/ml) (Invitrogen), 20μg/ml gentamicin (Gibco BRL Life Technologies), and 10% fetal bovine serum (Gibco). On the third and fifth day, 10 mls of fresh medium were added to the plate with growth factors, and the BMDC were collected at day 7 for phagocytosis assays.

The J774 (MØ) macrophage is a lineage culture cell derived from a reticulum cell sarcoma that has been extensively used to study yeast cell phagocytosis (35). J774 obtained from the ATCC were cultured in RPMI 1640, with 10% fetal calf serum (FCS; Vitrocell), 20 μg/ml gentamicin (Gibco BRL Life Technologies, Grand Island, NY, United States), and 1% nonessential amino acids in 90mm × 15mm Petri plate.

### Phagocytosis Assay

BMDC and MØ were plated at 10^7^ cells/plate (TPP, 75 cm^2^; 40 flasks of BMDC and 20 flasks of MØ). The BMDC and MØ used for experimentation displayed > 95% viability as determined by staining with Trypan blue. Cells were activated with IFN-*γ* (156 U/ml) for 12 hours prior to use in phagocytosis assays. Heat inactivated PL yeast (Pb8334) were added in the culture at a ratio of 5x1 (yeast x cells) and incubated for 8 hours at 5% CO_2_ and 37°C. After phagocytosis, the cells were removed from the flasks using a cell scraper, transferred to 50 ml falcon tubes and centrifuged for 10 min at 300 g at 4°C. Supernatants were discarded and pellets washed 2 times with PBS. Cells were lysed using buffer Tris [HCl (pH 7,6), 150mM NaCl com 1% de Igepal-CA630 (Sigma) supplemented with protease inhibitor (Roche)] for 2 hours at 4°C. After lysis, the samples were centrifuged for 10 min at 1,300g and 4°C. The supernatants were collected and centrifuged for 1 hour at 100,000g. The supernatants were collected and subjected to an immunoprecipitation assay.

### Immunoprecipitation Assay

The protocol of immunoprecipitation was performed using Protein G (Dynabeads Protein G Immunoprecipitation Kit; Thermo Fisher Scientific). Briefly, the anti-MHC-II antibody (Clone KH74 and AF6-120.1; BioLegend) was linked to magnetic beads in the proportion 50 µg antibody to 200 µl of beads. To avoid the elution of the complex MHC-II + peptide, the beads were crosslinked with 5 mM BS3 diluted in Buffer Conjugation (20mM Na3PO4, 0.15M NaCl, pH 7.9). The Crosslink was stopped using Quenting Buffer (1 M Tris HCl, pH 7.5), and the beads were washed 3 times with PBS with 0,02% of Tween20. After placing the sample in a magnetic bookcase (DynaMag™-2, Invitrogen by Thermo Fisher Scientific) the supernatant was discarded and anti-MHC-II was added to the sample beads. The tube was removed from the magnetic and the sample was incubated for 30 min with agitation at room temperature. After incubation, the tube was again placed in the magnetic bookcase. The supernatant was discarded, and the pellet washed 3 times with Washing Buffer of the kit. After the last wash, the tube was removed from the magnetic, the supernatant discarded and Elution Buffer (50mM glycine, pH 2,8) was added to elute of the MHC-II-Peptide complex. The sample was incubated for 2 min with agitation at room temperature, placed into the magnetic bookcase and then the supernatant was collected for analysis by mass spectrometry.

### Mass Spectrometry and Data Analysis

The samples generated by the immunoprecipitation were analyzed in the Proteomic Unit of the Department of Biochemistry of the Institute of Chemistry of the Federal University of Rio de Janeiro (UFRJ). Samples were trypsinized (Promega), desalted by C-18 spin column (Havard Apparatus) and dried in a vacuum centrifuge. The peptides resulted were resuspended in 0.1% formic acid and analyzed using an EASY-nLC system (Thermo Scientific) coupled to LTQ - Orbitrap Velos mass spectrometer (Thermo Scientific). The samples loaded onto a precolumn (ReprosilPur C18, 2 cm × 150 μm i. d. × 5 μm) with a flow rate of 5 μl/min and separated on the analytical column (ReprosilPur C18, 30 cm × 75 μm i.d. × 1.7 μm) with a gradient from 100% mobile phase A (0.1% FA) to 34% phase B (0.1% FA, 95% ACN) during 60 min, 34%–95% in 15 min and 5 min at 95% phase B at a constant flow rate of 250 nL/min. The LTQ-Orbitrap Velos was operated in positive ion mode with data-dependent acquisition. The full scan was obtained in the Orbitrap with an automatic gain control (AGC) target value of 10e6 ions and a maximum fill time of 500 ms. Each precursor ion scan was acquired at a resolution of 60,000 FWHM in the 400–1500 m/z mass range. Peptide ions were fragmented by CID MS/MS using a normalized collision energy of 35, target value of 2e5 ions and maximum fill time of 100ms. The 20 most abundant peptides were selected for MS/MS and dynamically excluded for a duration of 30s.

The raw data was processed with Proteome discoverer 2.1 software (Thermo Scientific). Peptides identifications were performed using the Sequest HT algorithm against FASTA format databases, contained protein sequences of PL and Mus musculus (for exclusion) provide by Uniprot (https://www.uniprot.org/). The searches were performed with peptide mass tolerance of 10 ppm, MS/MS tolerance of 0.5 Da, tryptic cleavage specificity, 2 maximum missed cleavage sites, fixed modification of carbamidomethyl (Cys), oxidation (Met), acetyl (N-terminus) and phospho (Ser, Thr, Tyr). False discovery rates (FDR) were obtained using Target Decoy PSM selecting identifications with a q-value equal or to less than 0.01.

### *In Silico* Prediction

For in silico prediction, we used MHC-II Binding Predictions (http://tools.iedb.org/mhcii/) software from the Immune Epitope Database and Analysis Resource (IEDB) (http://www.iedb.org/). This software enables predictions of interactions of our peptide structures with MHC-II on host cells. The predictions for each protein allow for the ranking of the binding capacity of each peptide characterized by mass spectrometry. This rank, as a percentage, is a mean between the prediction methods available in the database. A peptide with a rank less than 10% is considered likely to be presented by MHC-II (36). The three-dimensional (3-D) representation of the proteins and the location of the peptides in the proteins was achieved using I-Tasser (http://zhanglab.ccmb.med.umich.edu/I-TASSER/) and the figures were generated using PyMol version 2.3 software (http://pymol.org/2/).

### Peptides Synthesis

The peptides predicted to efficiently bind MHC-II were chemically synthesized by Biomatik Corporation (Ontario, CA) with purity of ≥95% as determined by HPLC and mass spectrometry.

### Presentation of Peptides to BMDC and MØ

To study the ability of BMDC and MØ to present the peptides, BMDC or MØ in the concentration of 10^6^ cells/mL were seeded into 96-well plates in RPMI medium with 5, 10 or 20 µg/mL of each peptide or without peptide for 6h in CO_2_ at 37°C. After the incubation, cells were collected, stained with florescent antibody and analyzed by flow cytometry.

### BMDC and MØ Flow Cytometer Analyze

BMDC and MØ incubated with or without the peptides (5, 10 or 20 µg/mL) were washed with FACS buffer twice. Then, supernatants were removed and FACS buffer with Live/Dead – BV450 (BD Horizon); MHC-II – PE-Cy7 (clone: M5/114.15.2; Biolegend); F4/80 – FITC (clone: BM8; Biolegend); CD11c – BV711 (clone: N418; Biolegend); CD80 – PE (clone: 16-10A1; eBioscience); CD86 – APC (clone: GK1.5; Biolegend) were added for immunophenotyping and assessment of activation. The cells were incubated for 30 min at 4°C in the dark, washed 2X with FACS buffer and fixed with 1% paraformaldehyde. Sample acquisition was performed on a flow cytometer FACS BD LSRII housed in the Flow Cytometry Core Facility at the Einstein.

### Lymphoproliferation Assay

We performed two different experiments to assess for lymphoproliferation. In the first approach, BALB/c mice were immunized *via* the subcutaneous route with two different mixtures of 4 peptides (Mix1: LBR1, LBR2, LBR3, LBR4; Mix2: LBR5, LBR6, LBR7, LBR8) at the final concentration of 10 µg/ml of each peptide in Freud’s complete adjuvant. After 7 days of immunization the mice were euthanized, the spleens and lymph nodes were harvested. Splenocytes and lymphocytes were incubated 90x16 culture plate in RPMI 1640 for 2h at 37°C to enrichment of the lymphocytes. After incubation, non-adherent cells were collected and stained with 5 μM CFSE in sterile PBS with 2% of FBS at room temperature in the dark for 15 min. The labeling was stopped with cold FBS and the samples were incubated for 5min at 4°C in the dark. Then, the cells were washed 2X with RPMI 1640 and suspended in complete RPMI medium (1% non-essential amino acids, 1% sodium pyruvate, 10% Fetal Bovine Serum, 2mM L-glutamine, 50μM b-mercaptoethanol, 50μg/ml ciprofloxacin). The lymphocytes were counted in Neubauer’s chamber and 2x10^5^ cells were plated in 96-well plate. After plating, the lymphocytes were stimulated or not with 10µg/ml of each peptide separately or the lymphocytes were co-cultured with DCs (5 lymphocytes: 1 DC) with or without each peptide. The cultures were incubated in CO_2_ at 37°C for 72h, and then the supernatants collected and frozen at -20°C for subsequent cytokine analyses. The lymphocytes and the lymphocytes in co-culture with DCs were stained with Live/Dead – BV450; MHC-II – PE-Cy7; B220 – PE-Cy7; CD3 – PE; CD4 – APC; CD8 – PerCP and CSFE – FITC and T cell proliferation was evaluated by flow cytometry using a FACS BD LSRII.

In the second approach, BALB/c mice were infected intratracheally (i.t.) with log phase PL (Pb8334) yeast that were maintained on Sabouraud agar for 7 days at 37°C. To prepare the inoculum used in the experiments, PL was harvested from the tubes and washed three times with phosphate buffered saline (PBS, pH 7.2). Thereafter, large and agglutinated yeast cells were separated by decanting, and the small isolated yeast cells were collected and counted by hemocytometer, yeast cells with a viability of >95% as determined by Trypan blue staining. For the procedure, animals were anesthetized intraperitoneally using ∼200μl of a solution containing 80 mg/kg ketamine and 10 mg/kg xylazine (both from União Química Farmacêutica, Brazil). Five minutes after the injection of the anesthetics, a small longitudinal skin incision was made in the neck to expose the trachea and 1x10^7^ yeast cells in 50 μl of PBS was injected. The incision was sutured with 5-0 silk. After 15 days of infection the mice were euthanized, the spleens and lymph nodes were harvested. Splenocytes and lymphocytes were incubated 90x16 culture plate in RPMI 1640 for 2h at 37°C to enrichment of the lymphocytes. Then, the cells were washed 2X with RPMI 1640 and suspended in complete RPMI. The lymphocytes were counted in Neubauer’s chamber and 2x10^5^ cells were plated in 96-well plate. After plating, the lymphocytes were stimulated or not with 10µg/ml of each peptide separately or the lymphocytes were co-cultured with DCs (5 lymphocytes: 1 DC) with or without each peptide. The cultures were incubated in CO_2_ at 37°C for 72h, and then the supernatants collected and frozen at -20°C for subsequent cytokine analyses.

### Cytokine Detection by Cytometric Bead Array (CBA) and ELISA

For cytokine determinations, the supernatants from the lymphoproliferation assays were thawed and analyses were performed using the BD CBA Mouse Th1/Th2/Th17 Cytokine Kit (BD Bioscience, San Jose, CA, USA), which measures IL-2, IL-4, IL-6, IFN-γ, TNF-α and IL-17A. The samples were measured on a FACS BD LSRII and analyzed by FCAP Array 3 Software (BD Bioscience). The theoretical limits of detection for the kit Th_1_/Th_2_/Th_17_ are 0.1 pg/mL for IL-2, 0.03 pg/mL for IL-4, 1.4 pg/mL for IL-6, 0.5 pg/mL for IFN-γ, 0.9 pg/mL for TNF and 0.8 pg/mL for IL-17A.

For cytokine detection in the supernatant from the lymphoproliferation assay after the mice were infected with PL, we thawed the samples and assayed with the IL-2, and IFN-γ using ELISA kits (BD OpTeia, San Diego, CA, United States). The detection limits of the assays were as follow: 3.1 pg/ml for IL-2, 31.3 pg/ml for IFN-γ as determined by the manufacturer.

### Statistical Analysis

All assays were performed at least twice on separate dates using triplicates. The murine immunization assays were performed on two independent dates with two groups (Mix1 and Mix2) using 5 mice per group each time. GraphPad Prism 8 software (San Diego, CA, United States) was used to run the statistical analysis. The results were expressed as mean values and standard deviations (SD) of the indicated values. The data were previously analyzed as regards normality by Shapiro-Wilk’s method and then One-way ANOVA with Tukey’s post-test and confirmed by “t” test were employed for parametric data. p values of ≤0.05 were used to indicate statistical significance.

## Results

### Mass Spectrometry Assay

Mass spectrometry analysis identified 18 PL peptides from the cultures of BMDCs and/or macrophages (MØ) with heat-inactivated PL (Pb8334) yeast (**Table 1**). The identified peptides were associated with 15 proteins and their respective genes (**Table 1**). After protein identification, we use the FASTA format of PL deposited in the Uniprot (https://www.uniprot.org/) and MaxQuant software (https://www.maxquant.org/) to perform *in silico* predictions to determine percent rank for peptide binding to MHC-II according to the Immune Epitope Database and Analysis Resource (IEDB) consensus method described above. The *in-silico* prediction identified only one peptide (ITVTSQSAFDAQFNR) with a rank lower than 10% (**Table 1**).

**Table 1:** Proteins and peptides characterized by mass spectrometry after being presented by BMDC and/or MØ, and their IEDB rank.

aa Quantitate	Sequenc	Protein name – Access name	Presented by:	IEDB Rank (%)
**14**	VPPAIAQFQNTLDR	60S ribosomal protein L8-B –PAAG_04998	BMDC	63.5%
**11**	NFGIGQDIQPK			65.0%
**16**	LKVPPAIAQFQNTLDR			54.0%
**11**	QVHPDTGISNR	Histone H2B – PAAG_08918	BMDC	40.5%
**15**	AMSILNSFVNDIFER			63.5%
**12**	DAGTIAGLNVLR	Hsp70-like protein –PAAG_01262	BMDC	28.1%
**25**	QHPSELETAIAGALSDLEANTPDLK	40S ribosomal protein S7 – PAAG_07182	BMDC	22.0%
**11**	VNIGQILLSVR	60S ribosomal protein L10-B –PAAG_01052	BMDC	28.5%
**11**	KVLTIINANQR	Ribosomal protein L35 –PAAG_02889	BMDC	16.0%
**17**	AIGIQPTEEGTIAVTTK	Ribosomal_L28e domain-containing protein –PAAG_03664	BMDC	51.5%
**15**	LESGNFSWGSEGISR	40S ribosomal protein S8 –PAAG_00264	BMDC	92.0%
**27**	NAALTVGNILPLGSVPEGTVVTNVEEK	60S ribosomal protein L2 –PAAG_00430	BMDC	61.0%
**15**	ITVTSQSAFDAQFNR	H15 domain-containing protein – PAAG_12131	BMDC	9.7%
**13**	IPYFNAPIYLENK	H/ACA ribonucleoprotein complex subunit –PAAG_05102	BMDC	53.5%
**16**	EDATALLYADPHNPMR	SET domain-containing protein – PAAG_12069	BMDC/MØ	54.5%
**11**	RFVNVTLTGGK	40S ribosomal protein S30 – PAAG_00206	BMDC	40.5%
**21**	ENGCIIFISGLEDGTLLVCSK	tRNA ligase – PAAG_02801	BMDC/MØ	90.0%
**17**	GPRDHPFYSMSTQADGK	Uncharacterized protein –PAAG_12107	BMDC	38.5%

In addition to synthesizing and testing the peptide with the IEDB less than 10%, we also generated two peptides located in the protein Histone H2B, one peptide located in the Hsp70-like protein, two peptides 60S ribosomal protein for which three peptides were characterized, and two other peptides that showed IEDBs of 16 and 22%. The peptides selected were named LBR1, LBR2, LBR3, LBR4, LBR5, LBR6, LBR7 and LBR8 (**Table 2**).

**Table 2:** Peptides selected to be synthesized and how they were named.

Protein name – Access name	IEDB Rank (%)	Peptide named/Sequence
**60S ribosomal protein L8-B –PAAG_04998**	65.0%	LBR1/LKVPPAIAQFQNTLDR
	54.0%	LBR2/NFGIGQDIQPK
**Histone H2B – PAAG_08918**	40.5%	LBR3/QVHPDTGISNR
	63.5%	LBR4/AMSILNSFVNDIFER
**Hsp70-like protein – PAAG_01262**	28.1%	LBR5/DAGTIAGLNVLR
**40S ribosomal protein S7 – PAAG_07182**	22.0%	LBR6/QHPSELETAIAGALSDLEANTPDLK
**Ribosomal protein L35 – PAAG_02889**	16.0%	LBR7/VNIGQILLSVR
**H15 domain-containing protein – PAAG_12131**	9.7%	LBR8/ITVTSQSAFDAQFNR

### 3-D Prospecting of Protein

**Figure 1:**
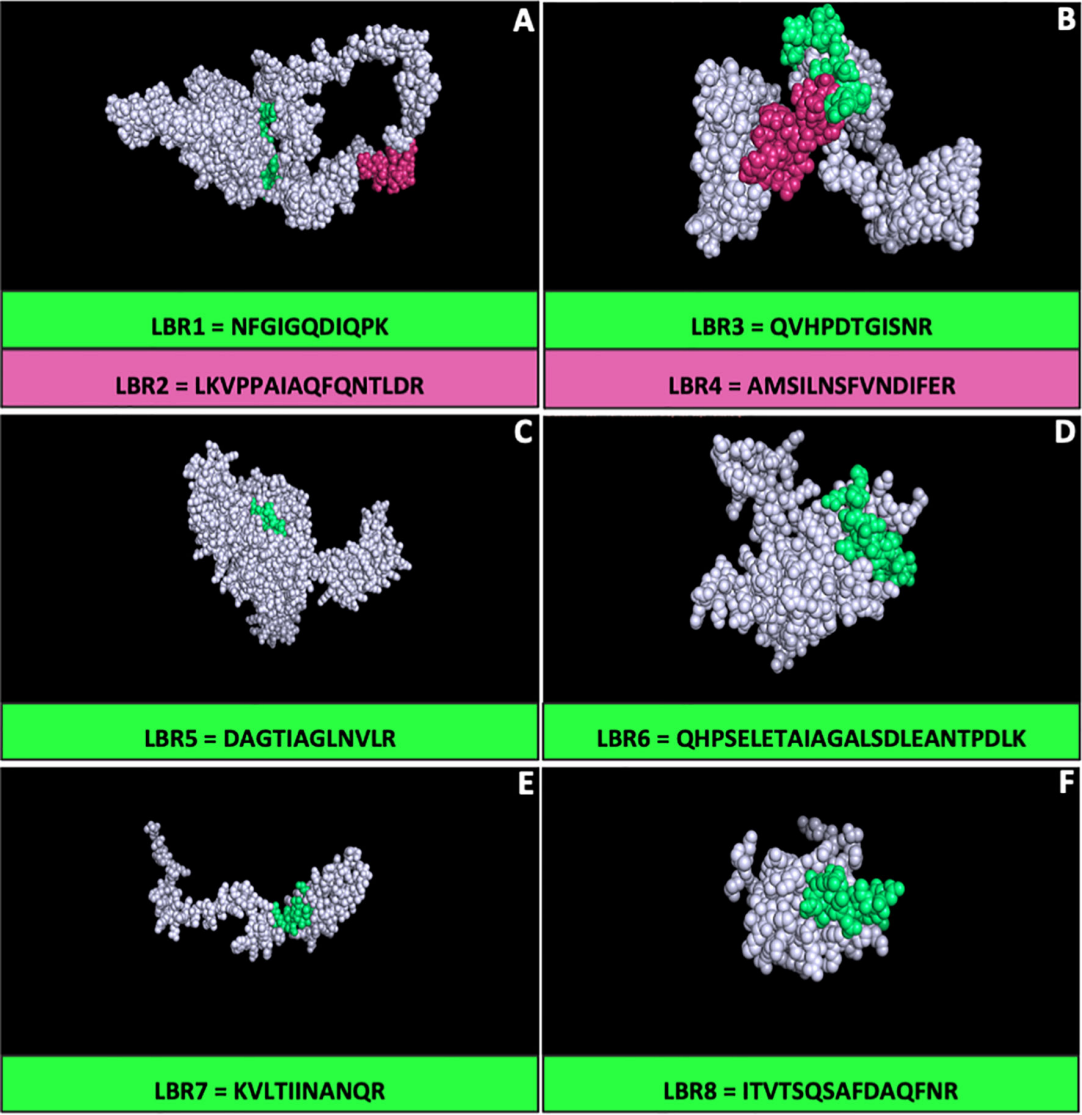
Three-dimensional (3-D) model of the proteins of *Paracoccidioides lutzii* by I-TASSER. The models were generated using peptides identified using immunoproteomics and the generated structure of their respective proteins. The figures and the peptide position in the protein were visualized using the PyMol software for **(A)** 60S ribosomal protein L8-B –PAAG_04998; **(B)** Histone H2B – PAAG_08918; **(C)** Hsp70-like protein – PAAG_01262; **(D)** 40S ribosomal protein S7 – PAAG_07182; **(E)** Ribosomal protein L35 – PAAG_02889; **(F)** H15 domain-containing protein – PAAG_12131.

All proteins that had their respective peptides synthetized were analyzed three-dimensionally (3-D) to characterize the peptide positions in these proteins using the I-Tasser platform and the figures were visualized using the PyMol version 2.3 software (**Figure 1**). The positions of each peptide were evaluated by 3-D modeling, particularly to determine whether they were presented on the surface. LBR2, LBR3, LBR4, LBR6 and LBR8 were the most exposed peptides.

### Peptide Ability to Stimulate BMDC and MØ

**Figure 2:**
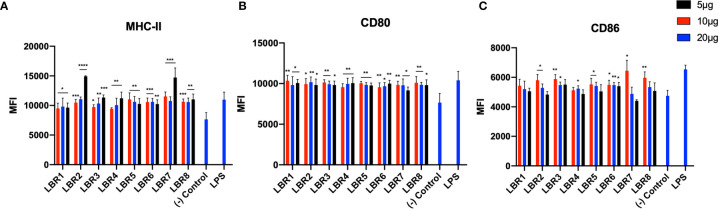
The activation profiles of BDMC after being pulsed with peptides were analyzed by the mean fluorescence intensity (MFI) of the MHC-II **(A)**, CD80 **(B)** and CD86 **(C)** molecules in the cells wall. These results are from three independent experiments. Data were analyzed by one-way ANOVA followed by Tukey’s post-test and confirmed by “t” test, where *p < 0.05, **p < 0.01, ***p < 0.001, ****p < 0.0001 in comparison to (-) Control group (culture of BMDC without stimulus).

**Figure 3:**
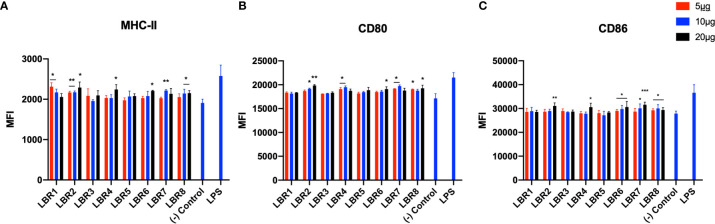
The activation profiles of MØ after being pulsed with the peptides were analyzed by the mean fluorescence intensity (MFI) of the MHC-II **(A)**, CD80 **(B)** and CD86 **(C)** molecules in the cells wall. These results are from three independent experiments. Data were analyzed by one-way ANOVA followed by Tukey’s post-test and confirmed by “t” test, where *p < 0.05, **p < 0.01, ***p < 0.001 and ****p < 0.0001 in comparison to (-) Control group (culture of MØ without stimulus).

BDMC and MØ were pulsed with different concentrations of each peptide (5, 10 or 20µg/mL) for 6h (**Figures 2** and **3**) and the cells were analyzed by flow cytometer to evaluate if the peptides increased the expression of costimulatory molecules on the cell membrane. Controls were untreated or LPS exposed. For BMDC, we observed that all peptides significantly increased the expression of MHC-II and CD80 molecules (**Figures 2A, B**). LBR1, LBR3, LBR5, LBR6 and LBR8 peptides also enhanced the expression of CD86 molecules (**Figure 2C**). The concentration of 10µg/mL of peptide generated the most consistent results (**Figure 2**).

Pulsing of MØ with LBR2, LBR4, LBR6, LBR7 or LBR8 significantly increased the expression of the CD80 and CD86 (**Figures 3B, C**). The expression of MHC-II (**Figure 3A**) significantly increased when MØ were pulsed with LBR1, LBR2, LBR4, LBR6, LBR7 or LBR8. Again, the concentration of 10µg/mL produced the most consistent results (**Figure 3**).

In comparing the data between the BDMC and MØ, LBR2, LBR4, LBR6, LBR7 and LBR8 were the peptides that showed the best ability to stimulate both BMDC and/or MØ. Given the consistency of results with 10µg/mL peptide, we chose this concentration for murine immunization.

### Lymphoproliferation Assay

**Figure 4:**
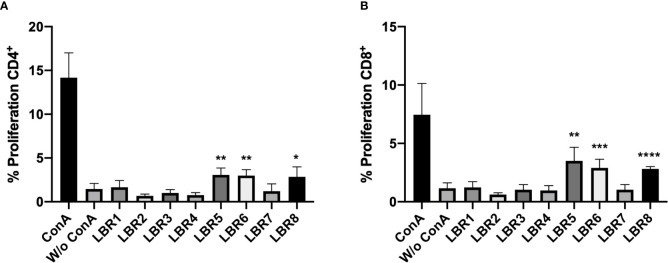
Proliferation of CD4^+^ **(A)** and CD8^+^ **(B)** T cells. Lymphocytes obtained from mice immunized with the mix of peptides (Mix1: LBR1, LBR2, LBR3, LBR4 or Mix2: LBR5, LBR6, LBR7, LBR8) were incubated with peptides for 72 h and T cell proliferation was analyzed by flow cytometry using the CFSE dilution method. Lymphocytes cultivated in the absence of a stimulus (W/o ConA) were used as a negative control and lymphocytes cultivated with Concanavalin A were used as positive control (ConA). The data shown are from two independent experiments. Data were analyzed by one-way ANOVA followed by Tukey’s post-test and confirmed by “t” test, where *p < 0.05, **p < 0.01, ***p < 0.001 and ****p < 0.0001 in comparison to unstimulated lymphocytes (W/o ConA).

CD3^+^CD4^+^ and CD3^+^CD8^+^ T cells obtained from spleens and lymph nodes of mice after immunization with the peptides were subjected to proliferation assays. The LBR5 and BLR6 peptides significantly stimulated the proliferation of CD4^+^ T cells (**Figure 4A**), while CD8^+^ T Cells showed statistically increased proliferation when treated with LBR5, LBR6 or LBR8 peptides (**Figure 4B**).

**Figure 5:**
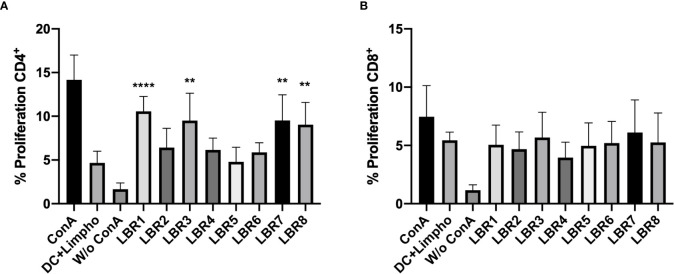
Proliferation of CD4^+^ **(A)** and CD8^+^ **(B)** T cells. BMDCs pulsed or not with a peptide were co-cultured with lymphocytes from mice immunized with the mix of peptides (Mix1: LBR1, LBR2, LBR3, LBR4 or Mix2: LBR5, LBR6, LBR7, LBR8) for 72 h and T cell proliferation was analyzed by flow cytometry using the CFSE dilution method. Lymphocytes cultivated in the absence of stimulus (W/o ConA) were used as negative control, lymphocytes cultivated with Concanavalin A were used as positive control (ConA) and co-cultures with BMDCs without peptide and lymphocytes without ConA (DC+Limpho) were the control groups. The data shown are from two independent experiments. Data were analyzed by one-way ANOVA followed by Tukey’s post-test and confirmed by “t” test, where **p < 0.01 and ****p < 0.0001 in comparison to co-culture of BMDCs and lymphocytes without stimulus (DC+Lympho).

We then assessed the ability of BMDC to present peptides to CD3^+^CD4^+^ and CD3^+^CD8^+^ T Cells (**Figure 5**). In this system, BMDCs pulsed with the LBR1, LBR3, LBR7 or LBR8 significantly enhanced the proliferation of co-cultured CD4^+^ T cells when compared to unstimulated BMDCs (**Figure 5A**). CD8^+^ T cells proliferation was similar between co-cultures of unstimulated BDMCs and BMDCs pulsed with the peptides (**Figure 5B**).

### Cytokine Production

**Figure 6:**
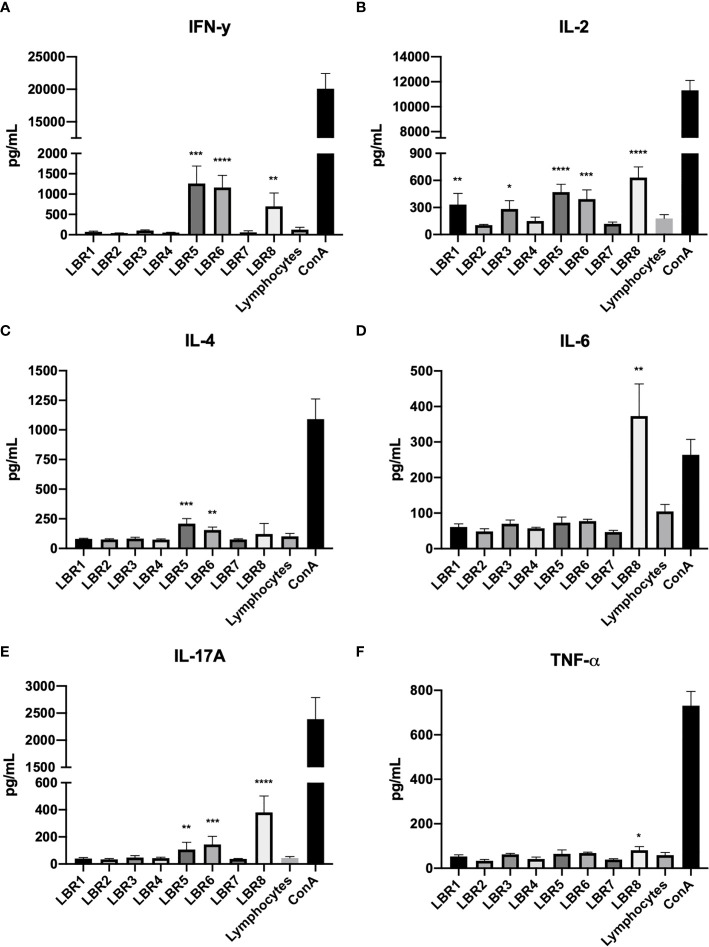
Cytokine profiles in the supernatants of lymphoproliferation with the peptides (IFN-γ, IL-2, IL-4, IL6, IL17A and TNF-α cytokines represented in **A–F** respectively). The supernatants obtained from the lymphoproliferation after 72 h of incubation with the peptides were analyzed for cytokine levels by CBA. The supernatant from the lymphocytes cultivated in the absence of any kind of stimulus (Lymphocytes) was used as a negative control and the supernatant from the lymphocytes cultivated with Concanavalin A was used as a positive control (ConA). The data shown are from two independent experiments. Data were analyzed by one-way ANOVA followed by Tukey’s post-test and confirmed by “t” test, where *p < 0.05, **p < 0.01, ***p < 0.001 and ****p < 0.0001 in comparison to supernatant from the lymphocytes without stimulus (Lymphocytes).

Cytokine concentrations of supernatants from the first approach of lymphoproliferation with the peptides and the co-cultures with BMDCs pulsed with the peptides were determined (**Figure 6**). In the supernatants from the lymphoproliferation with the peptide LBR8, we observed significant increases of IFN-γ, IL-2, IL-6, IL-17A and TNF-α (**Figures 6A, B, D–F**, respectively). LBR5 and LBR6 stimulated the significant increase of IFN-γ, IL-2, IL-4 and IL-17A (**Figures 6A–C, E**, respectively) and we also observed the significant increase of IL-2 in the supernatants from assays with LBR1 and LBR3 (**Figure 6B**).

**Figure 7:**
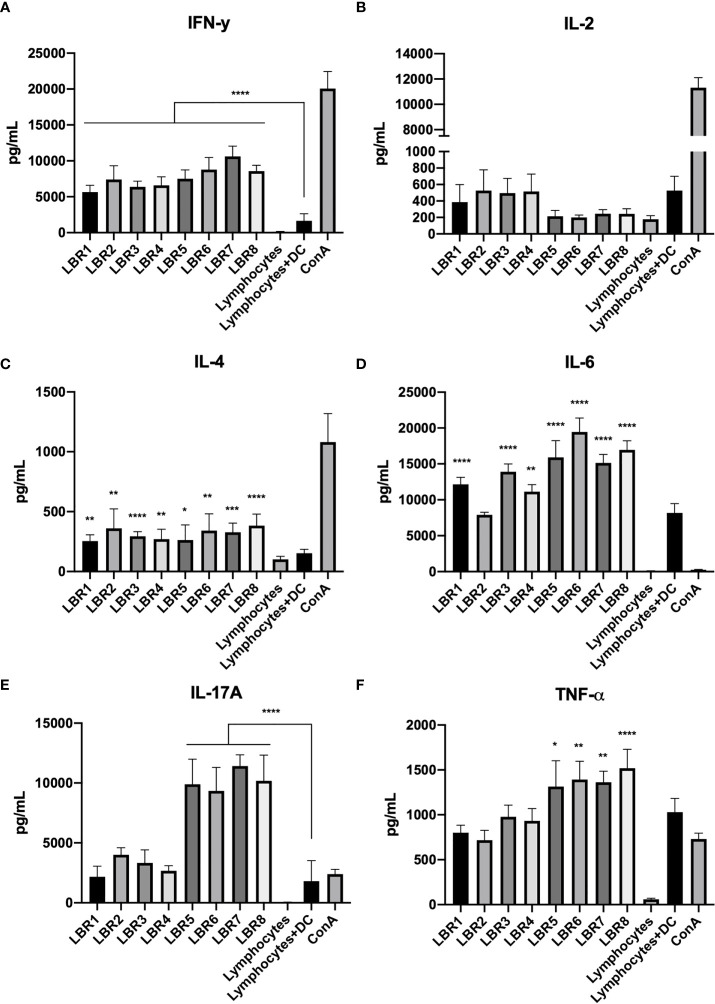
Cytokine profiles in the supernatant of co-culture lymphoproliferation (IFN-γ, IL-2, IL-4, IL6, IL17A and TNF-α cytokines represented in **A–F** respectively). The supernatants come from the co-cultures of lymphocytes (from peptide immunized mice) and BMDCs pulsed with peptides and proliferation was analyzed for cytokine levels by CBA. Lymphocytes cultivated in the absence of stimulus (Lymphocytes) were used as negative control, lymphocytes cultivated with Concanavalin A were used as positive control (ConA), and co-culture with BMDCs without be pulsed with peptide and lymphocytes without ConA (DC+Limpho) were the control groups. The data shown are from two independent experiments. Data were analyzed by one-way ANOVA followed by Tukey’s post-test and confirmed by “t” test, where **p < 0.01 and ***p < 0.001 in comparison to co-culture of BMDCs and lymphocytes without stimulus (DC+Lympho).

Cytokine measurements were also performed with supernatants obtained from the lymphocytes in co-culture with BMDCs pulsed with the peptides (**Figure 7**). IL-4 and IFN-γ concentrations were increased in the supernatants from all conditions with peptide stimulation (**Figures 7A, C**). IL-6 was similarly increased, except for the condition with LBR2 (**Figure 7D**). Interestingly, IL-2 did not increase with exposure to any of the peptides (**Figure 7B**). TNF-α and IL-17A levels increased significantly in the LBR5, LBR6, LBR7 and LBR8 co-culture supernatants (**Figures 7E, F**).

**Figure 8:**
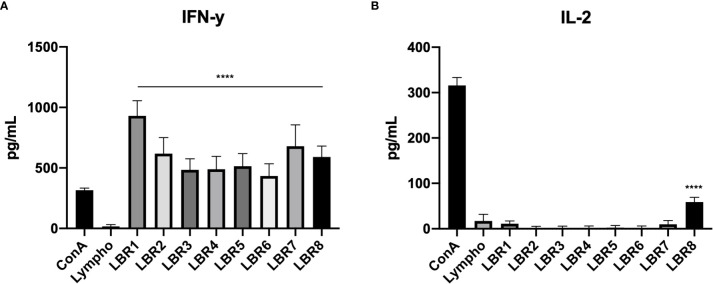
Cytokine profiles in the supernatants of lymphocytes from infected mice with the peptides (IFN-γ and IL-2 cytokines represented in **A, B** respectively). The supernatants obtained from the lymphoproliferation after 72 h of incubation with the peptides were analyzed for cytokine levels by ELISA. The supernatant from the lymphocytes cultivated in the absence of any kind of stimulus (Lymphocytes) was used as a negative control and the supernatant from the lymphocytes cultivated with Concanavalin A was used as a positive control (ConA). The data shown are from two independent experiments. Data were analyzed by one-way ANOVA followed by Tukey’s post-test and confirmed by “t” test, where ****p < 0.0001 in comparison to supernatant from the lymphocytes without stimulus (Lymphocytes).

Measurements of cytokine levels were also performed with supernatant from lymphoproliferation of lymphocytes from PL-infected mice (**Figure 8**), which showed significant increases of IFN-γ in the supernatant from all conditions with peptide stimulation when comparted with the control group which was the culture of lymphocytes only (Lympho) (**Figure 8A**). However, IL-2 levels increased significantly only when lymphocytes were exposed to LBR8 (**Figure 8B**).

**Figure 9:**
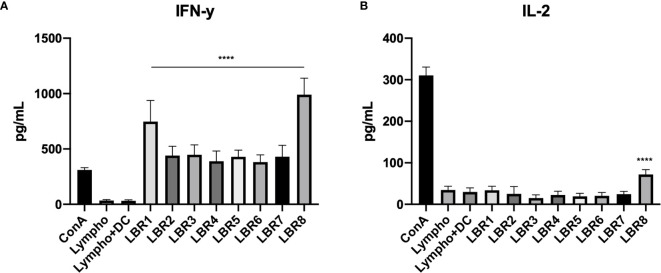
Cytokine profiles in the supernatant of co-culture lymphoproliferation (IFN-γ and IL-2 cytokines represented in **A, B** respectively). The supernatants come from the co-cultures of lymphocytes from infected mice and BMDCs pulsed with peptides and cytokine levels were analyzed by ELISE. Lymphocytes cultivated in the absence of stimulus (Lympho) were used as negative control, lymphocytes cultivated with Concanavalin A were used as positive control (ConA), and co-culture with BMDCs without be pulsed with peptide and lymphocytes without ConA (Limpho+DC) were the control groups. The data shown are from two independent experiments. Data were analyzed by one-way ANOVA followed by Tukey’s post-test and confirmed by “t” test, ****p < 0.0001 in comparison to co-culture of BMDCs and lymphocytes without stimulus (Lympho+DC).

Cytokine measurements were also performed with supernatant from lymphoproliferation of co-culture of lymphocytes from mice infected with DCs pulsed with peptides (**Figure 9**). Results similar to those found in the supernatant of lymphoproliferation with peptides in the co-culture of lymphocytes from mice infected with DCs pulsed with peptides supernatant were observed. We observed significant increases of IFN-γ in the supernatant from all condition with DCs pulsed with peptides when compared with the control group, which were co-cultures of lymphocytes and DCs without peptide (Lympho + DC) (**Figure 9A**). IL-2 increased significantly only in response to LBR8 (**Figure 9B**).

## Discussion

PCM is one the most important systemic mycosis in the Latin America, and the rates of infection with *Paracoccidioides* species have been increasing steadily over the past few years (7), especially within the new agricultural borders in Brazil, which are considered endemic to PL, in particular (9–12). The treatment of PCM presents several challenges that complicate effective patient care, including prolonged treatment time and a high incidence of relapses. Hence, there is an urgent need to identify new therapeutic approaches to combat this complex disease. One of the most promising treatment innovations for PCM is the inclusion of the peptide P10 in therapy, which is highlighted by P10’s ability to be used both early in or after disease in immunologically normal or compromised mice (21–24, 26). However, P10 does not sufficiently correspond to sequences in PLGp43. Hence, we sought to identify immunostimulatory peptides in PL that could be developed as therapeutics. During the performance of this project, we use bioinformatic and immunoproteomic tolls, they are potentially useful tool to identify disease-associated antigens that can be used to elicit the immune responses or in the diagnostic, both methodologies has been currently used in the paracoccidioimycosis context and they are presenting promisor results (28, 37).

In this study, we identified peptides from PL with a capacity to stimulate T cell responses using an immunoproteomic assay followed by *in silico* prediction. We found 18 peptides that met predictive criteria for having a capacity to be presented by MCH-II from BMDC and/or MØ. Three of these peptides came from the same protein [60S ribosomal protein L8-B, a protein synthase that is suppressed during phagocytosis (38)], which may suggest that this protein can be quite immunogenic. Another fact that may suggest that this protein can be immunogenic is that it is located in the fungal cell wall (39, 40). Two peptides were derived from Histone H2B, which is also found in the cell wall (39, 40). In *Histoplasma capsulatum*, cell wall H2B has been targeted with mAb’s, and opsonization by mAb to H2B alters the intracellular fate of the yeast and results in protection in a murine model of lethal histoplasmosis (41). One peptide is derived from Hsp70. Hsp70 is one of the highly recognized antigens by antibodies in sera of patients with either histoplasmosis or PCM, confirming the fungal expression and immunogenicity of HSP70 during infection (42–47). Peptides LBR1, LBR2, LBR3, LBR4, LBR5, LBR6, and LBR7 came from protein that have previously been identified in the fungal cell wall by Silva, R.P. and collaborators (40). LBR8 belongs to the Histone H1 protein, which is located in the cell nucleus, as describe in the UniProt.

The 8 peptides with the lowest IEDB ranks or derived from known cell wall expressed proteins were synthesized and tested *in vitro* to determine their ability to stimulate BDMC and MØ, and their capacity to stimulate lymphocytes proliferation was assessed. The PL peptides increased the expression of key superficial molecules (MCH-II, CD80 and CD86) on BDMC and MØ, which is consistent with a capacity of the peptides to change the activation of these cells. This is consistent with our findings with P10, which similarly increased the expression of these markers on BDMC (22–24). We also found that lymphocytes from mice immunized with a mix (Mix1 or Mix2) of peptides in Freund’s adjuvant proliferated significantly in response to each individual peptide. Similarly, DCs pulsed with each peptide efficiently stimulated lymphocyte proliferation. Specifically, peptides LBR5 and LBR6 induced CD4^+^ and CD8^+^ T lymphocyte proliferation and LBR8 induced CD8^+^ T lymphocyte proliferation. DCs pulsed LBR1, LBR2, LBR7 or LBR8 induced CD4^+^ T lymphocyte proliferation. Cytokine analyses revealed that these proliferative responses were characterized by a mix of Th1, Th2 and Th17 T cells, since we observed the production of IFN-γ, IL-2, IL-4, IL-6, TNF-α and IL-17A.

Immunoprotection in PCM is mediated by Th1/Th17 responses that are controlled by the anti-inflammatory activity of Treg cells (48). In contrast, severe forms of disease are associated with Th-2/Th-9 responses and excessive expansion of Treg cells. Our result demonstrated that LBR5, LBR6, and LBR8 promoted a mixed Th1, Th2, and Th17 response. However, LBR5, LBR6 and LBR8 induced increased levels of pro-inflammatory cytokines (IFN-γ, IL-2, IL-6, TNF-α and IL-17A), which are consistent with a robust Th1 immune response that especially promotes cellular proliferation and the formation of granulomas (IFN-γ and TNF-α) (22, 24, 49). Th17 immunity was also induced, as demonstrated by increased concentrations of IL-6 and IL-17A (50–52). Hence, our findings suggest that these peptides have the potential to be utilized in the treatment of PCM due to PL. They may also be useful in the diagnosis of the disease.

Remarkably, each of the PL peptides tested was able to induce proliferation of lymphocytes primed by previous immunization of mice with the peptides LBR5, LBR6 and LBR8. Calich and collaborators (53) in a susceptible-resistant experimental model correlated the specific antibody response and the polyclonal B-cell activation with progressive infection in the susceptible B10.A mice whereas resistant A/Sn mice developed a strong DTH response when challenged with PB with low levels of specific antibodies and no evidence of B cell activation (53). Taborda et al. (20) identified that the peptide (P10) as the immunodominant T cell epitope in PBGp43 as it was able to induce proliferation, including that of lympho-node CD4^+^ T cells of the Th1 subtype proliferation, forming INF-γ and IL-2 (20). In the present work, we found that lymphocytes from mice infected with PL (PB8334) and stimulated with LBR8 had cytokine profiles suggesting proliferation with significant increase of IL-2. We observed an immune response by CD4^+^ T cells subtype Th1 with production of IFN-γ in response to all 8 peptides. These results are similar to those found by Taborda and collaborators in 1998, when P10, the most promisor peptide to be used in the PCM treatment to date, was identified (20). These results suggest that the peptide LBR8 as well as the other immunostimulating peptides may also be applied therapeutically. Future studies will explore the effectiveness of the PL peptides as vaccines and examine whether the PL proteins associated with these peptides can be used as diagnostics.

## Data Availability Statement

The datasets presented in this study can be found in online repositories. The names of the repository/repositories and accession number(s) can be found below: https://www.uniprot.org/, A0A0A2V091, https://www.uniprot.org/, C1GPI5, https://www.uniprot.org/, C1HDS5, https://www.uniprot.org/, C1H2V9, https://www.uniprot.org/, A0A0A2V0A9, https://www.uniprot.org/, C1GP19, https://www.uniprot.org/, C1GXU1, https://www.uniprot.org/, C1GRW7, https://www.uniprot.org/, C1H2K5, https://www.uniprot.org/, C1GNW1, https://www.uniprot.org/, C1H8U1, https://www.uniprot.org/, C1GRA7, https://www.uniprot.org/, C1GWA6, https://www.uniprot.org/, C1GWJ4.

## Ethics Statement

All procedures were performed according to the guidelines of National Council of Ethics with Animals (CONCEA) and the protocols were approved by the Ethical Committee for Animal Use from Institute of Biomedical Sciences at University of Sao Paulo (CEUA ICB USP certificates 101/2014, approved in 01/12/2014) and at Albert Einstein College of Medicine (Einstein) under the animal use protocol n° 00001281.

## Author Contributions

LS performed the experiments, analyzed the data, and wrote the manuscript. CT and LC performed proliferation experiments. MM and MJ performed the immunoproteomic assay. JN and CT conceived the project, designed the experiments, and wrote and revised the manuscript. All authors contributed to the article and approved the submitted version.

## Funding

This research was funded by Fundação de Amparo à Pesquisa do Estado de São Paulo (FAPESP), grants number 2016/08730-6, 2018/25171-6 and 2019/20622-2. CNPq (grant number 420480/2018-8), Coordenação de Aperfeiçoamento de Pessoal de Nível Superior – CAPES.

## Conflict of Interest

The authors declare that the research was conducted in the absence of any commercial or financial relationships that could be construed as a potential conflict of interest.
